# Enhanced Formability of Magnesium Alloy Rolled Plates by $\left\{10\bar{1}2\right\}$ Tensile Twinning and Recrystallization

**DOI:** 10.3390/ma15186253

**Published:** 2022-09-08

**Authors:** Jiafei Deng, Jing Tian, Yancai Zhou, Yuanying Chang, Wei Liang, Jinyao Ma

**Affiliations:** 1 College of Materials Science and Engineering, Taiyuan University of Technology, Taiyuan 030024, China; 2 Shanxi Key Laboratory of Advanced Magnesium-Based Materials, Taiyuan University of Technology, Taiyuan 030024, China; 3 Instrumental Analysis Center, Taiyuan University of Technology, Taiyuan 030024, China

**Keywords:** twin variant, DDRX, CDRX, TDRX, texture

## Abstract

To solve the problem of poor formability of magnesium alloys, the bending and straightening process was used to successfully introduce large-volume $\left\{10\bar{1}2\right\}$ tensile twins and dynamic recrystallization into the plates, and the comprehensive mechanical properties of the plates were improved, in which the anisotropy index (Lankford value: $\bar{r}$) decreased by 77%, and the corresponding Erishen value (IE) increased by 88%. The research shows that most of the continuous dynamic recrystallization (CDRX) and discontinuous dynamic recrystallization (DDRX) inherit the grain orientation of the parent grains, and a few have deviations from the parent grains. The twinning-assisted dynamic recrystallization (TDRX) can effectively inherit the grain orientation of the parent grain and retain the orientation relationship of the $\left\{10\bar{1}2\right\}$ tensile twin. The cooperation of the pre-set tensile twinning and various dynamic recrystallization processes leads to the deflection of the basal plane, which effectively weakens the basal texture and promotes the activation of various non-basal slip systems. Combined with grain refinement strengthening and dislocation strengthening, the magnesium alloy plate, after bending and straightening, obtains good comprehensive mechanical properties.

## 1. Introduction

As the lightest metal structural material, magnesium alloys can produce lightweight products. Due to their high specific strength, specific stiffness, and good damping and shock absorption, they have made great progress in the manufacturing process of lightweight automobiles [1,2,3,4]. However, the poor formability of magnesium alloys is the reason for the difficulties faced in manufacturing complex components. It is mainly because the components are mostly in the state of tensile stress during the formation process; therefore, their formability is poor. Therefore, how to improve the formability of magnesium alloys to realize the industrialized production and application of magnesium alloy complex components is the current research hotspot [5,6,7,8,9].

Stamp formability can usually be assessed by the Erickson test, which is related to the elongation and plastic strain ratio. Song et al. found that a low anisotropy index (Lankford value) can improve the formability of magnesium alloy plates [10]. Therefore, the way to improve the room temperature formability of magnesium alloy plates is to reduce the Lankford value of the material during plastic deformation.

Rolled magnesium alloys usually form a strong basal texture of the grain c-axis//ND (normal direction), resulting in large plate anisotropy and poor formability [11,12,13]. Researchers have carried out extensive research on the problem of the poor stamping formability of magnesium alloys. The research shows that the weakening of basal texture can weaken the anisotropy of magnesium alloy plates and significantly improve the formability of plates [12,14].

Twinning plays an important role at low temperatures and when the grain size is large or the strain rate is high [15]. Although twinning cannot dominate plastic deformation at large strains, deformation twinning can cause a complete reorientation of the crystal that has twinned, which results in substantial texture modification. The twinning modifies the texture to induce slip generation to coordinate the plastic deformation of magnesium alloys. Among them, the $\left\{10\bar{1}2\right\}$ tensile twinning is easily activated at room temperature, due to its low critically resolved shear stress and leads to a deflection of about 86.3° in the basal, which is regarded as an effective means to weaken the basal texture.

Xin et al. performed compression deformation on the width of a rolled magnesium alloy plate by pre-rolling and introducing twinning deformation [16]. Huo et al. obtained finer grains and weak and random texture by wave bending forming [17]. Song and Hong et al. found that the formability of magnesium alloy plates can also be improved by the pre-twinned reorientation texture [10,14]. Kim et al. achieved the introduction of $\left\{10\bar{1}2\right\}$ tensile twins in magnesium alloy plates using a die [18,19].

However, in the current research, the improvement of stamping formability of magnesium alloy plates used to prepare complex components is still insufficient. Moreover, the effects of pre-set tensile twinning and various recrystallization behaviors on the basal plane texture are still not fully explored.

Based on this, the bending and straightening processing technology is adopted in this paper, which not only ensures the improvement of the properties of the magnesium alloy plate but also ensures that the stress is be more uniform during the deformation process and that the unstable fracture will not occur. The formability of the plate was detected by testing and calculating the Lankford value of the AZ31 magnesium alloy plate. At the same time, a large volume of $\left\{10\bar{1}2\right\}$ tensile twins were preset in the plate, which effectively weakened the basal texture, and the effects of twinning behavior and various dynamic recrystallization behaviors on the basal texture are deeply analyzed. Establishing the relationship between the microstructure evolution and mechanical properties of magnesium alloy plates during bending and straightening has guiding significance for the improvement of the room temperature formability of magnesium alloys.

## 2. Material and Methods

### 2.1. Material Processing

**Figure 1:**
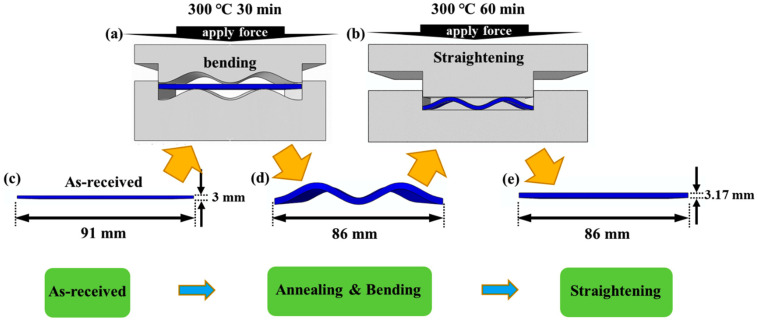
Bending and straightening process: (**a**) bending mold, (**b**) straightening mold, and (**c**–**e**) morphology of the plate at different stages of bending and straightening.

In this paper, the initial material used for this work was a commercial hot-rolled AZ31 magnesium alloy plate, with a thickness of 3 mm. The magnesium alloy plate was cut into billets, with a plane dimension of 91 mm (rolling direction (RD)) × 86 mm (transverse direction (TD)). Using the bending and straightening process, the plate is compressed along the RD to preset $\left\{10\bar{1}2\right\}$ tensile twins. The specific process is shown in Figure 1.

The original plate is first placed in a bending mold (the mold is designed with a cosine curve, as shown in Figure 1a). The plate and mold were kept at 300 °C for 30 min to eliminate residual internal stress and balance the structure, and to prevent grain growth caused by a long holding time. Then, it was bent and deformed, and the deformed plate is shown in Figure 1d. The bent plate was placed in a straightening mold (Figure 1b). Since dynamic recrystallization occurs in the temperature range of 150–450 °C, the straightening process is carried out at 300 °C to ensure plastic deformation of magnesium alloys and to help to obtain a large number of tensile twins. After the mold and plate were kept at 300 °C for 60 min, the straightening process was performed to obtain a plate with an RD length of 86 mm (Figure 1e). The strain in the RD is about 5.5%, and the loading force is 3 MPa.

To better distinguish the states of magnesium alloy plates at different stages during the whole bending and straightening process, the microstructure and properties of the as-received sample, the sample after annealing at 300 °C for 30 min, the sample after annealing and bending, and the sample after straightening were analyzed.

### 2.2. Mechanical Property Test

The as-received sample, the sample after annealing, and the sample after straightening were subjected to uniaxial tensile tests in three directions (RD, 45° (45° angle with RD), TD) at room temperature, and we measured their mechanical property curves and Lankford value (r-value). The size of the sample was 45 mm (RD) × 6 mm (TD) × 3 mm (ND), and the test was carried out using a CMT5250 computer-controlled electronic universal testing machine (10 N–10 kN). The extensometer gauge length was 15 mm and the tensile speed was 0.5 mm/min. In this paper, some tensile tests were interrupted at 7% strain to obtain Lankford values. In addition, the Lankford values were calculated according to ISO 6892–1:2009 and ISO 10113:2006 standards. Erichsen tests were performed to determine the formability of AZ31 Mg plates using a BTP-600 microcomputer-controlled plate forming tester, with a diameter of 20 mm. Each condition was repeated 3 times to obtain representative results.

### 2.3. Material Characterization

The Rigaku Smart Lab SE diffractometer was used to detect the macrotexture of the magnesium alloys in different states, and the sample size was 15 mm (RD) × 15 mm (TD) × 3 mm (ND), which was taken from the vicinity of the tensile specimen. A field emission scanning electron microscope (SEM; S8000G), equipped with EDS and electron backscatter diffraction (EBSD) systems, was used to observe the RD-TD planes of the specimens to characterize the microstructure of different samples. The EBSD samples were sequentially ground with 25 μm, 10 μm, 5 μm, 3 μm, 1.5 μm, and 0.5 μm grit sandpaper until the surface was smooth, and the ground samples were electropolished with 5% perchloric acid and 95% alcohol solution. The electrolysis temperature was between −20 °C and −40 °C, the electrolysis time was 1 min, the electrolysis voltage was 45 V, and the current was about 0.35 A. The surface of the samples after electropolishing was bright and free of deep pits. To obtain a better EBSD measurement indexing rate, the step size was set to 0.5 μm. The obtained EBSD data were imported into Channel 5 software (Oxford Instruments NanoAnalys, Oxford, UK) for analysis.

## 3. Results and Analysis

### 3.1. Microstructure Evolution

**Figure 2:**
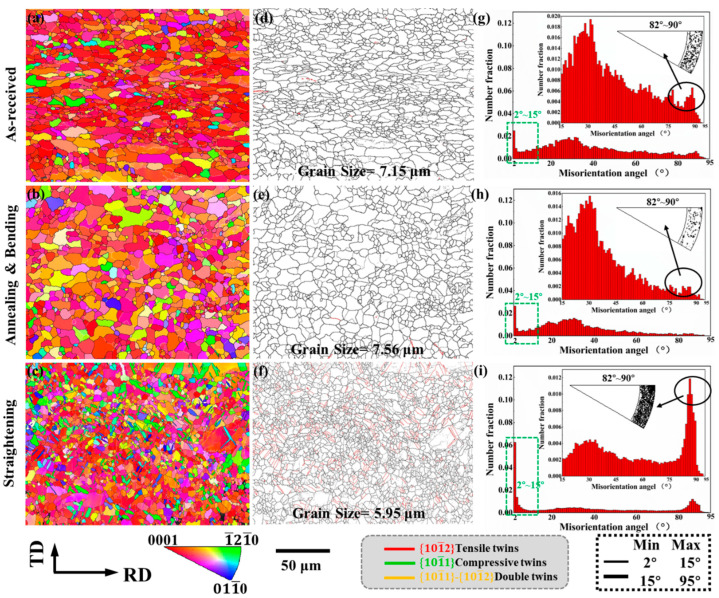
EBSD results: (**a**,**d**,**g**) as-received sample, (**b**,**e**,**h**) the sample after bending, and (**c**,**f**,**i**) the sample after bending and straightening; inserted in the figure is the distribution of misorientation rotation axes in the range of 82°~90°.

Figure 2 is the EBSD results of the as-received sample, the sample after annealing and bending, and the sample after straightening, using the IPF map, grain boundary map, and misorientation angle distribution to show the microstructure evolution of the sample at different stages of the bending and straightening process.

It can be observed from the figure that for the as-received sample, the c-axis of most of the grains is oriented towards ND, and the average grain size is 7.15 μm. After annealing at 300 °C for 30 min and bending, the area of misorientation angle distribution around 30° ± 5° increased, the recrystallized grains further increased, and the average grain size grew to 7.56 μm. There are almost no tensile twins in the as-received sample and the sample after annealing and bending. After straightening, the plate was pressed along RD, and a large number of tensile twins were preset. At the same time, the twins cut and refined the grains, and the average grain size was reduced to 5.95 μm.

In addition, it can be observed from the misorientation distribution (Figure 2g–i) that the content of low-angle grain boundaries (LAGBs) with misorientation angles in the range of 2°~15° increases after straightening. At the same time, from the distribution of the misorientation rotation axes in the range of 82°~90°, it can be observed that the $\left\{10\bar{1}2\right\}$ tensile twins are densely distributed after bending straightening.

**Figure 3:**
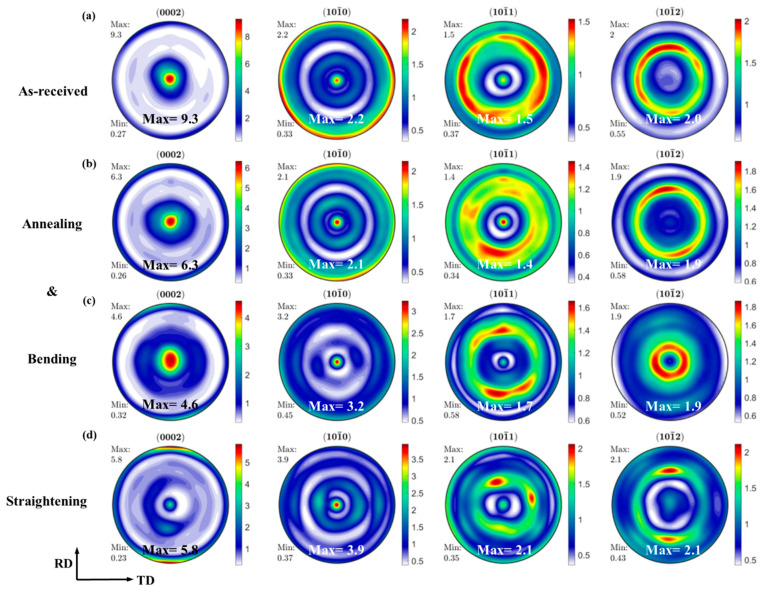
Macrotexture: (**a**) as-received sample, (**b**) the sample after annealing, (**c**) the sample after bending, and (**d**) the sample after bending and straightening.

Figure 3 shows the macrotexture results of different samples. The as-received sample has a typical strong basal texture of c//<0002>, with a texture strength of 9.3. After bending, the c-axis of the grains rotates to the RD, and the (0002) basal texture strength is further weakened to 4.6. The texture of the straightened plate forms an RD deflection texture; the basal texture is significantly weakened. In addition, the maximum texture is in the RD direction with a strength of 5.8. This is because the plate is compressed along the RD, and the c-axis of most grains rotates nearly 90° in the RD, resulting in the reduction in grains with basal plane orientation.

**Figure 4:**
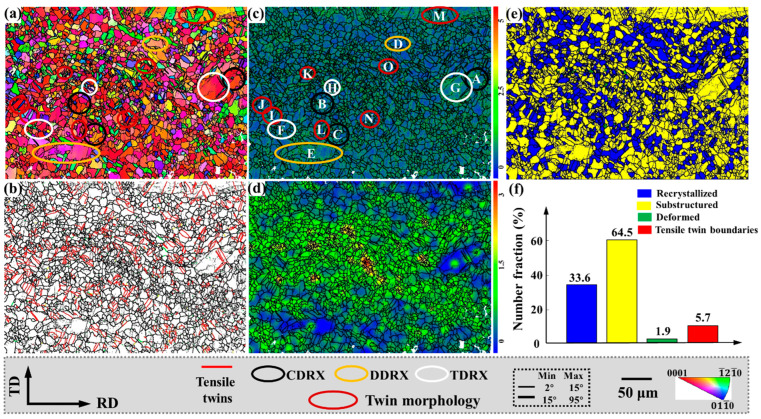
EBSD results of the sample after bending and straightening: (**a**) IPF, (**b**) tensile twin distribution, (**c**) the kernel average misorientation (KAM), (**d**) strain distribution, (**e**) the distribution, and (**f**) the content of different grains.

Figure 4 shows the IPF map, tensile twinning distribution, kernel average misorientation (KAM), strain distribution, and different microstructure distributions of magnesium alloy plates after bending and straightening.

The KAM distribution of Figure 4c reflects the distribution of the stored energy, while the stored deformation energy is proportional to the geometrically necessary dislocation density [20]. Therefore, the geometrically necessary dislocation density is higher around the tensile twin boundaries and LAGBs with higher KAM values. From the strain distribution diagram (Figure 4d), it can be observed that the regions with larger strains are all around the grains with smaller grain sizes, while the strains around the larger grains are smaller. Figure 4e,f show the distribution and content statistics of different structures. In the plate after bending and straightening, there are more cases of recrystallization, accounting for about 33.6% of the area, and a large number of tensile twins are produced. Both tensile twinning and recrystallization induce the c-axis deflection of the magnesium alloy grains, thereby weakening the basal texture. The grain orientation is more conducive to the activation of the slip system, which acts on the plastic forming of magnesium alloys.

### 3.2. Re-Orientation via DRX

The formation of dynamic recrystallization (DRX) texture contributes to the overall texture modification. It is well known that DRX behaviors mainly include continuous dynamic recrystallization (CDRX) [21] and discontinuous dynamic recrystallization (DDRX) [22].

In Section 3.1, we detected substantial dislocation activity in the KAM distribution, and it was reported that the high activity of dislocation slip during thermal deformation would lead to CDRX, as shown by the black circled area in Figure 4a. Furthermore, we observed obvious grain boundary bulges, as indicated by the yellow circled area, indicating the existence of DDRX.

#### 3.2.1. Continuous Dynamic Recrystallization (CDRX) Behaviors

**Figure 5:**
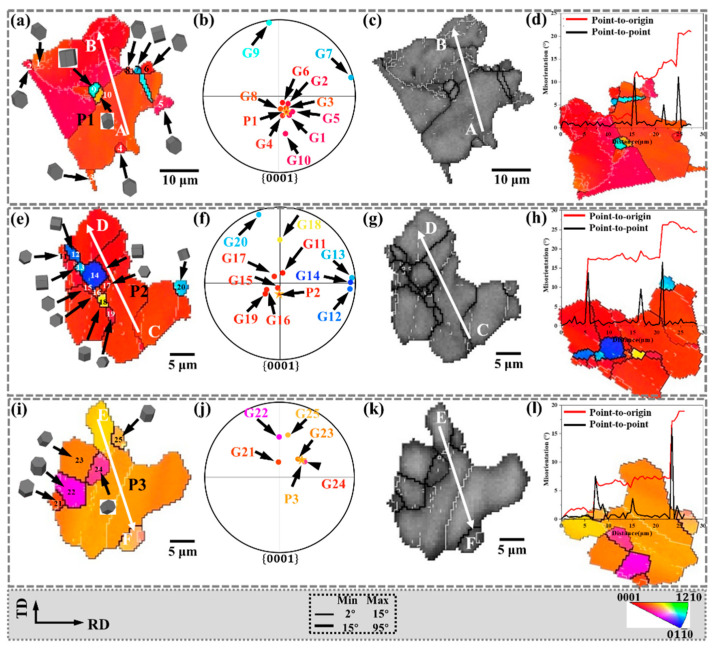
CDRX behavior of the black circle region in Figure 4 (**a**,**e**,**i**): the IPF, (**b**,**f**,**j**) {0001} pole figure, and (**c**,**g**,**k**) band contrast and (**d**,**h**,**l**) point-to-origin and point-to-point misorientations map along the black line AB, CD, and EF. The five-pointed star is the position of the parent grain on the {0001} pole figure.

To analyze the CDRX behavior, the regions A, B, C marked in Figure 4c are enlarged in Figure 5, and the corresponding IPF and band contrast (BC) maps are shown. The distribution of recrystallized grains in the {0001} pole figure is shown in Figure 5b,f,j, respectively. In addition, the white lines AB, CD, and EF are drawn in P1, P2, and P3, respectively. Furthermore, the corresponding in-grain misorientation angles are shown in Figure 5d,h,l, respectively.

The CDRX behavior is closely related to the accumulation of dislocations inside the grain [23]. Research shows that CDRX is always accompanied by high-density dislocation motion, intra-grain distortion, and subgrain boundary formation [24], and finally, new grains are formed. During this process, the LAGB is considered to be a subgrain boundary that continuously absorbs dislocations [25]. In Figure 5d,h,l, the LAGBs indicated that dislocations accumulated and transformed into subgrain boundaries, demonstrating the occurrence of CDRX.

In addition, the line from the point to the origin in Figure 5d shows an increasing trend, indicating that the misorientation angle increases along the AB line within P1. When the distance reaches 27.4 μm, the final misorientation angle is 21°, while the misorientation angle between the adjacent points is not significant. The point-to-origin curves in Figure 5h,l show similar trends, with final misorientation angles of 24.5° and 18.9°, respectively. The line from the point to the origin demonstrates the progressively increasing misorientation angle, which indicates the presence of substantial dislocation activity within P1, P2, and P3. Zhang et al. [26] observed a similar line profile in the hot-deformed AZ31 Mg alloy, and the point-to-origin misorientation angle increased to about 16°. Similarly, Pan et al. [27] also found an increasing trend in the point-to-origin misorientation angle. Both results indicated that CDRX occurred.

Both the LAGB and the progressively increasing misorientation angle along the line are important evidence for CDRX. In this process, part of the CDRX grain orientations inherit the parent grain orientation, and the parent grains are mostly basal plane orientations. The CDRX with the same grain orientation as the parent cannot change the basal texture. However, the other part of the CDRX will deviate from the grain orientation of the parent, which is beneficial to the weakening of the basal texture. Diverse grain orientations also have a positive effect on promoting the activation of the slip system.

#### 3.2.2. Discontinuous Dynamic Recrystallization (DDRX) Behaviors

**Figure 6:**
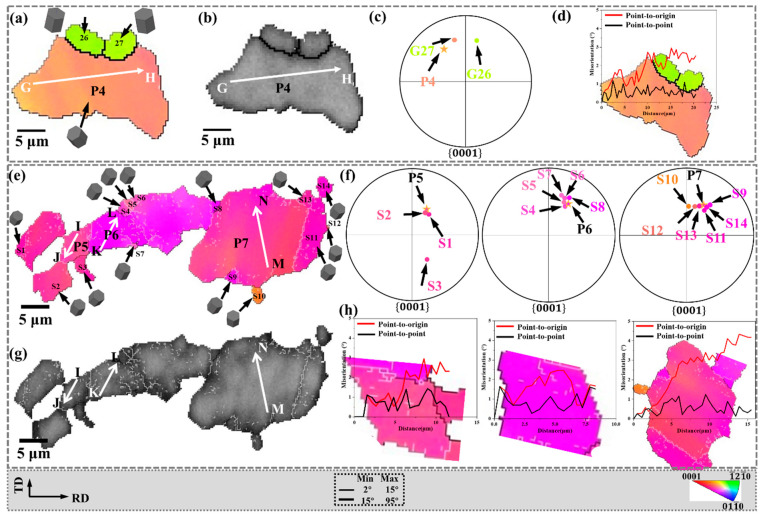
DDRX behavior of the yellow circle region in Figure 4: (**a**,**e**) the IPF, (**b**,**f**) {0001} pole figure, and (**c**,**g**) band contrast and (**d**,**h**) point-to-origin and point-to-point misorientations map along the black line GH, IJ, KL, and MN. The five-pointed star is the position of the parent grain on the {0001} pole figure.

In bending and straightening plates, DDRX behavior also exists. To facilitate analysis, the regions D and E marked in Figure 4c are enlarged in Figure 6. The corresponding IPF, BC, and {0001} pole figures are shown. The distribution of recrystallized grains in the {0001} pole figure is shown in Figure 6c,f, respectively. In addition, the white lines GH, IJ, KL, and MN are drawn in P4, P5, P6, and P7, respectively, and the corresponding in-grain misorientation angles are shown in Figure 6d,h, respectively.

DDRX typically involves the bulge of the original grain, followed by the formation of new grains [28], and HAGB migration is the dominant mechanism during high-temperature deformation [29]. This may be due to the higher mobility of grain boundaries at high temperatures. As shown in Figure 6a,b, the maximum misorientation angle accumulated along the line GH is not higher than 3.0°, indicating that the deformation inside P4 is negligible. However, the orientation difference between P4 and dynamically recrystallized grains (G26, G27) is quite large. This is because the dynamically recrystallized grains are formed by the migration of pre-existing HAGBs. Therefore, in Figure 6a,b, the bulge of HAGB is considered as strong proof of DDRX.

Furthermore, as shown in Figure 6e,g, the parent grains P5, P6, and P7 were transformed into subgrains (labeled as S1–S11), forming bulges separated by LAGB. These subgrains are expected to develop into new DRX grains [30]. The {0001} pole figure in Figure 6f shows that almost all of these subgrains (S1–S11) are distributed around the parent grains (P5–P7), indicating that they have similar grain orientations. Figure 6h shows the point-to-point and point-to-origin grain misorientation angles on the white line within the parent grains (P5–P7). The maximum misorientation angle of the point to the origin does not exceed 4.1°, indicating that the distortion inside the parent grain is relatively small.

The bulge of HAGB and the lower misorientation angle along the line indicate the behavior of DDRX. Part of the grain orientation of DDRX inherits the parent grain, and the other part deviates from the parent grain. In addition, when the orientation of the parent grains is not basal plane orientation, the formation of DDRX will promote the increase of non-basal plane orientation grains and play a further role in weakening the basal texture.

### 3.3. Texture Component of Tensile Twinning

In terms of the development of deformation texture in magnesium alloys, the deformation twinning produces the most significant texture changes. In addition, $\left\{10\bar{1}2\right\}$ tensile twinning has an extremely low CRSS (2–32 MPa) and is the most common twinning system in magnesium alloys. Tensile twinning, formed under tension along the c-axis or compression perpendicular to the c-axis [31], plays an important role in coordinating the strain along the c-axis in magnesium alloys [32,33]. There is an obvious positional relationship between the tensile twins after bending and straightening.

**Figure 7:**
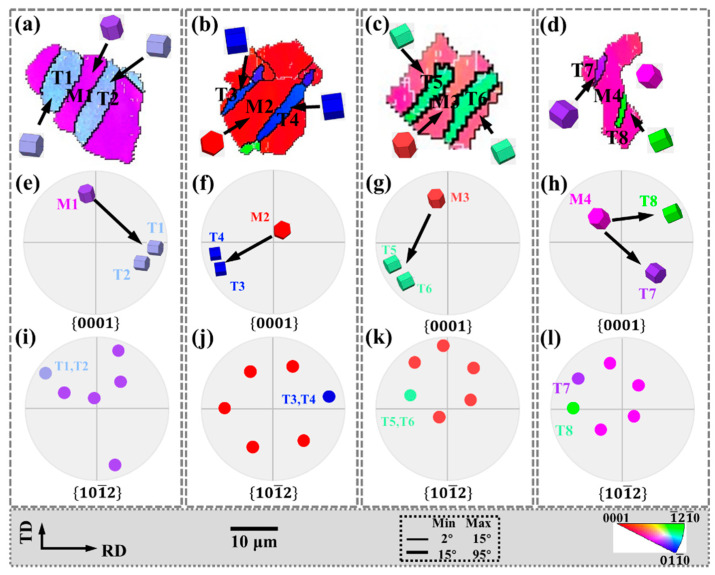
Parallel twin of the red circle region in Figure 4: (**a**–**d**) the IPF, (**e**–**h**) {0001} pole figure and (**i**–**l**) $\left\{10\bar{1}2\right\}$ pole figure.

Tensile twin variants of the same parent grain exist in an approximately “parallel” relationship. The grains in the regions I, J, K, and L in Figure 4c are enlarged in Figure 7, and the corresponding IPF, {0001} pole figures and $\left\{10\bar{1}2\right\}$ pole figures are also shown in Figure 7.

In Figure 7a–c, twin variants with the same grain orientation that are parallel to each other are activated in the parent grains (M1–M3). In the {0001} pole figure, the angle between the c-axis of the parent grain and the twin c-axis is approximately 86°, while the parallel tensile twins in the $\left\{10\bar{1}2\right\}$ pole figure are all the same variant. In Figure 7d, M4 activates twin variants with different crystal orientations that are parallel to each other are ortho variants in the $\left\{10\bar{1}2\right\}$ pole figure.

**Figure 8:**
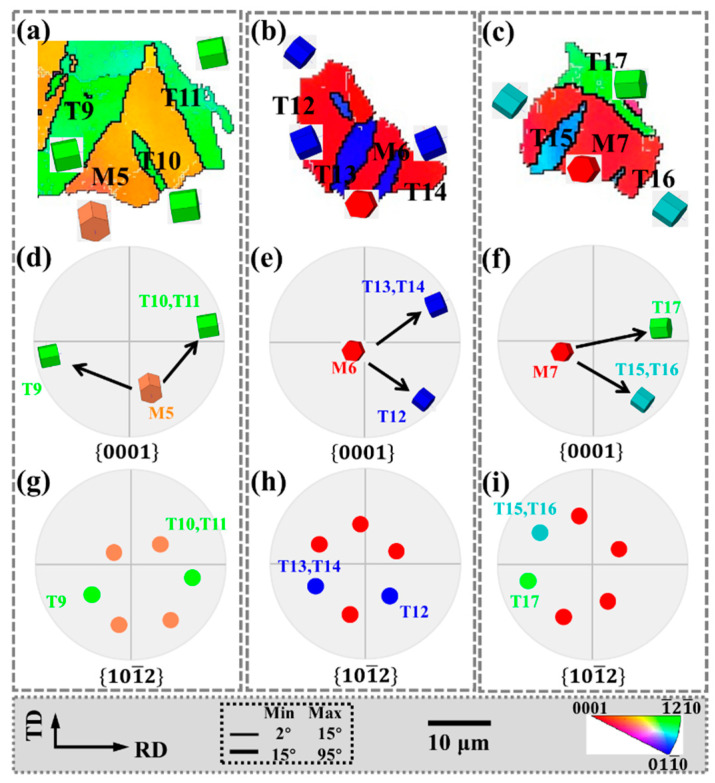
Non-parallel twin of the red circle region in Figure 4: (**a**–**c**) the IPF, (**d**–**f**) {0001} pole figure and (**g**–**i**) $\left\{10\bar{1}2\right\}$ pole figure.

Tensile twin variants with “non-parallel” relationships also exist in the same parent grain, as shown in Figure 8, which corresponds to the grains in the regions M, N, and O in Figure 4c. In Figure 8a, since T10 and T11 are approximately parallel and have the same grain orientation, the {0001} and $\left\{10\bar{1}2\right\}$ pole figures indicate that they are the same variant. However, although T9 and T10, T11 have the same grain orientation, they are approximately 45°, and the {0001} and $\left\{10\bar{1}2\right\}$ pole figures indicate that they are para-position variants. Similarly, in Figure 8b, since T13 and T14 are the same variants, T12, and T13, T14 have the same orientation but are approximately 120°. The {0001} and $\left\{10\bar{1}2\right\}$ pole figures show that they are spacer position variants. In Figure 8c, T15 and T16 are the same variants, which are approximately perpendicular to T17 but have different grain orientations. The {0001} and $\left\{10\bar{1}2\right\}$ pole figures indicate that they are neighbor variants.

Matrix grains that generate twins are mostly concentrated in the basal orientation, and the twins cause the basal to deflect at a large angle, which plays an important role in weakening the basal texture.

In addition, the twin boundaries around some twins are serrated, which is also characteristic of twinning-assisted dynamic recrystallization (TDRX) [34,35], as indicated by the white circles.

**Figure 9:**
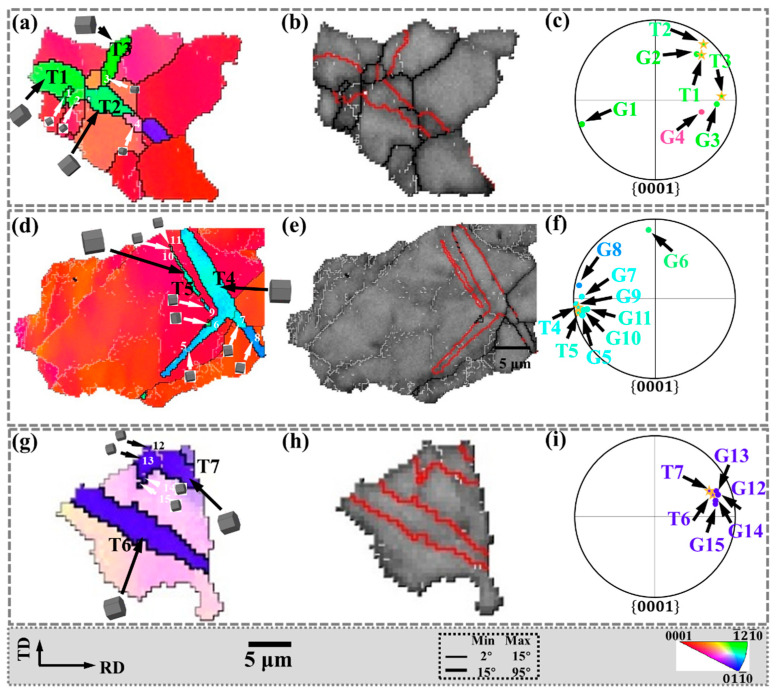
TDRX behavior of the white circle region in Figure 4: (**a**,**d**,**g**) the IPF, (**b**,**e**,**h**) band contrast and (**c**,**f**,**i**) {0001} pole figure. The five-pointed star is the position of the parent grain on the {0001} pole figure.

Except for CDRX and DDRX, with the formation of tensile twins, higher deformation energies are stored at the twin boundaries. DRX is easily activated around twins, as shown in Figure 9. The grains within the regions F, G, and H marked in Figure 4c are enlarged in Figure 9 and the corresponding IPF, BC, and {0001} pole figures are given. Among them, the tensile twin boundaries are identified in the BC diagram, and the distribution of recrystallized grains in the {0001} pole figure is shown in Figure 9c,f,i, respectively.

As shown in Figure 9c,f,i, some fine DRX grains (G1–G3, G5, G7, G9–G15) were observed in the twins (T1, T3–T5, T7) and almost all of these fine DRX grains are distributed around the twins (T1, T3–T5, T7) in the {0001} pole figure, indicating that they have similar grain orientations. In addition, DRX grains (G4, G6, G8) with other grain orientations are observed in some twins (T2, T4), and their distribution in the {0001} pole figure is quite different from that of the twins.

Figure 9a,d,g show that both LAGBs and HAGBs are observed in twinning (T1, T4, T7). It is shown that in the twins, due to the transformation of LAGBs into HAGBs, DRX eventually occurs, and new grains are formed in the twins. Meanwhile, the twin boundaries around the twins (T1–T7) are serrated. The above characteristics are classified as TDRX. 

After twin-induced dynamic recrystallization occurs, most of the new dynamic recrystallization can effectively inherit the grain orientation of the parent grain, retain the orientation relationship of twins, and act together with twins to weaken the basal texture.

### 3.4. Relationship between Twinning and Slip

**Figure 10:**
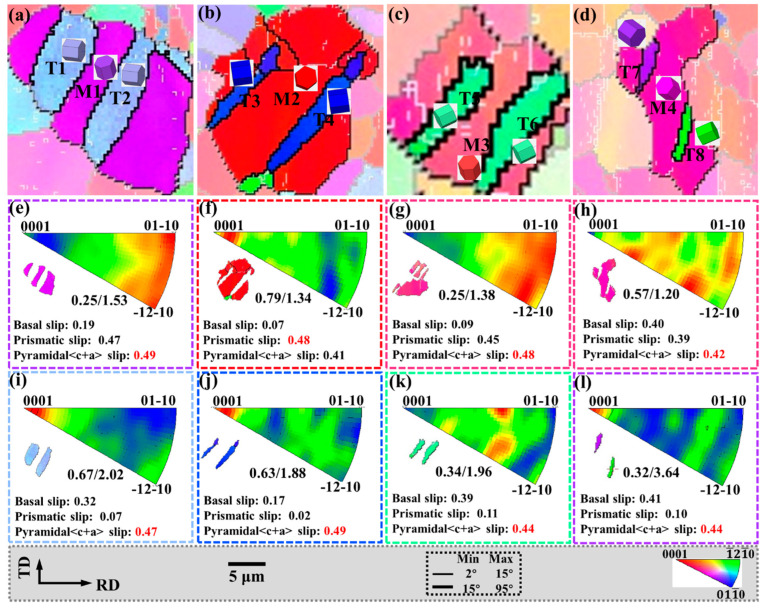
IGMA of parallel twins: (**a**–**d**) the IPF, (**e**–**h**) IGMA of the matrix, and (**i**–**l**) IGMA of the twins.

**Figure 11:**
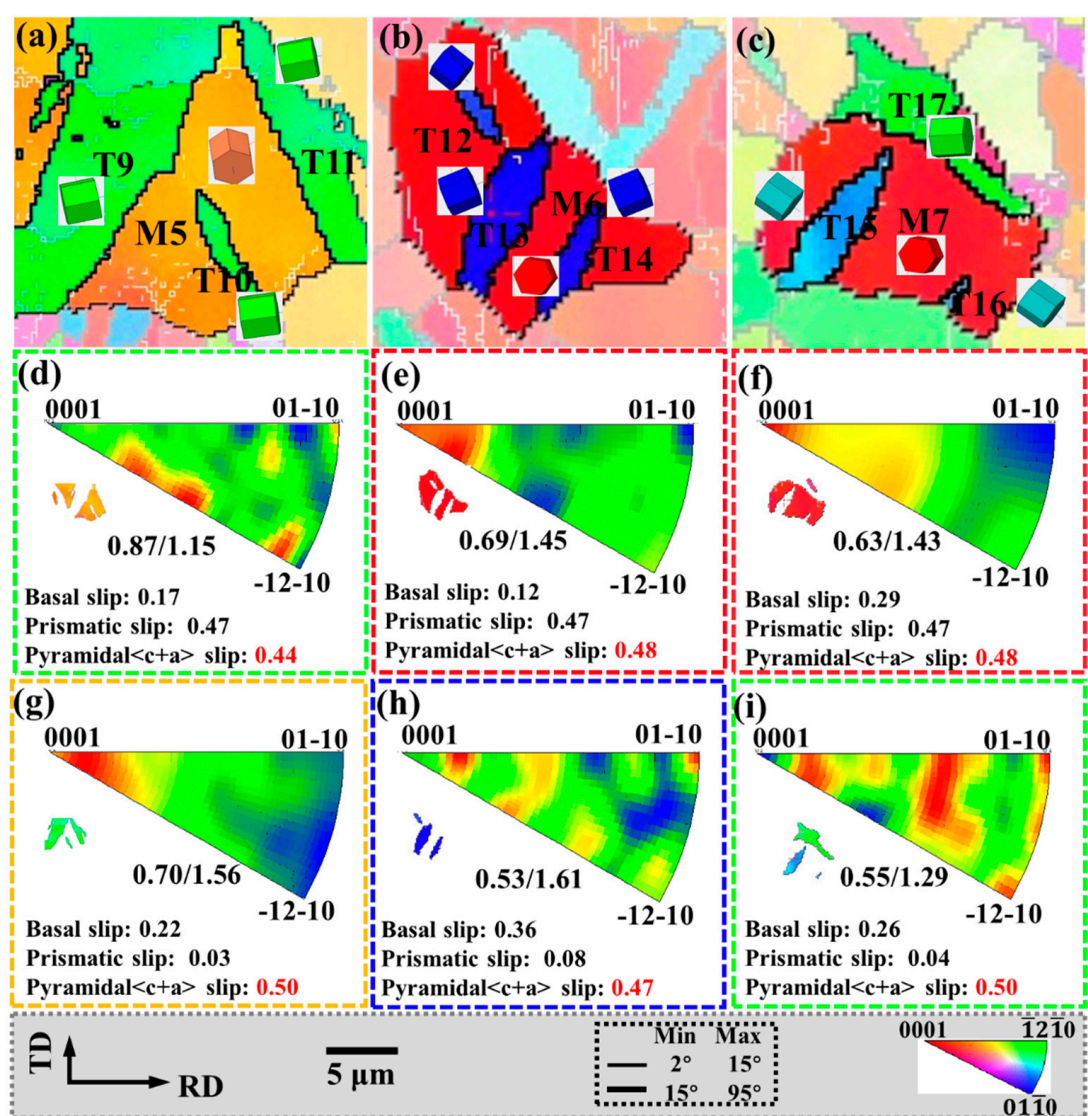
IGMA of non-parallel twins: (**a**–**c**) the IPF, (**d**–**f**) IGMA of the matrix, and (**g**–**i**) IGMA of the twins.

During the hot deformation of Mg alloys, dislocation slip can dominate the deformation under a large strain, although it has no effect on changing the texture [36]. Like twinning, slip plays an important role in coordinating the mechanical properties of magnesium alloys. To further investigate the interaction between twinning and non-basal slip during bending and straightening, the internal grain misorientation axis (IGMA) distributions of twinning and matrix within the regionals I-O are shown in Figure 10 and Figure 11, respectively. Activation of slip causes lattice rotation, and each slip system in magnesium alloys tilts around a fixed axis called the Taylor axis. When the lattice rotation distribution is concentrated around the <uvt0> axis, it indicates that the basal <a> slip or the pyramidal II<a+c> slip dominates. Prismatic <a> slip is more favorable for activation if the maximum intensity of IGMA in the matrix is rotated around the <0001> axis. When the IGMA distribution shows a relatively uniform distribution, the deformation process is caused by the joint activation of multiple slip systems [32,37].

For IGMA analysis, the misorientation angles involved in this work range from 0.5° to 2°; the lower misorientation angles are chosen to avoid errors due to the limited EBSD angular resolution, and the upper misorientation angles are chosen to eliminate misjudgment of other deformation mechanisms, such as shear bands [38]. To obtain accurate results, only the matrix and twins with over 500 measurement points were selected.

The IGMAs of M1 and M3 are all around the <uvt0> axis, and this distribution is caused by the activation of the basal <a> slip and the pyramidal II<a+c> slip. The Schmid factor of the pyramidal II<a+c> slip in these grains is the highest value, and the deformation temperature of 300 °C is enough to activate the pyramidal II<a+c> slip, so the activated slip system in the matrix is pyramidal II<a+c> slip. The IGMAs of M2, M6, and M7 are all around the <0001> axis, indicating that the prismatic <a> slip is activated in the matrix, and their Schmid factor values are also higher. In addition, the IGMA distributions of M4 and M5 are relatively uniform, indicating that various slip systems are activated in the matrix (Figure 10h and Figure 11d).

In Figure 10i,j,l, and Figure 11g, the IGMAs of all twins are around the <0001> axis, indicating that the prismatic <a> slip is activated in the tensile twins. In Figure 10k and Figure 11h,i, the IGMA distribution of the twins is relatively uniform, indicating that multiple slip systems are activated in the T5, T6, T16–T21 twins. Among them, the Schmid factor of the pyramidal II<a+c> slip is the highest value, and it is easier to activate during deformation.

During bending and straightening, the matrix grains activate a large amount of pyramidal II<a+c> slip and prismatic <a> slip to coordinate deformation, while more prismatic <a> slip is activated in the twin to accommodate the strain.

### 3.5. Mechanical Response after Bending and Straightening

**Figure 12:**
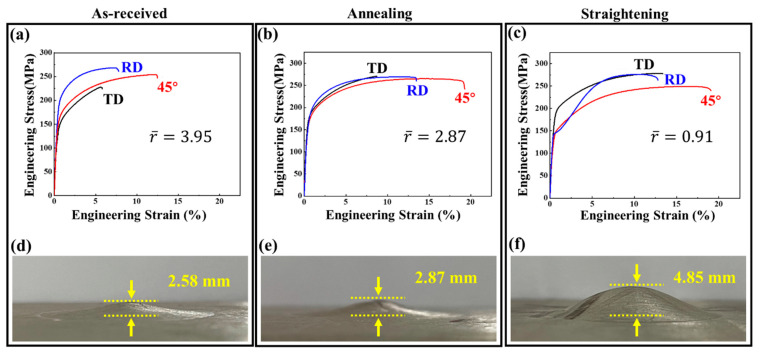
Mechanical behavior: (**a**,**d**) as-received sample, (**b**,**e**) the sample after annealing, and (**c**,**f**) the sample after bending and straightening.

Figure 12 shows the results of the mechanical properties of different samples stretched along the RD, TD, and 45° directions, respectively. Yield strength (YS), ultimate tensile strength (UTS), fracture elongation (FE), Lankford value (r-value), and Erishen value are shown in Table 1. The $\bar{r}$ value is based on the following formula:

(1) $$\bar{r}=\frac{r_{\mathrm{RD}}+2r_{45}+r_{\mathrm{TD}}}{4}$$

Grain size is significantly reduced due to twinned-cut grains and various dynamic recrystallization behaviors during high-temperature deformation (Figure 2). According to the Hall–Petch relationship [39], the lower the grain size, the better the grain refinement strengthening effect, and the higher the YS and UTS. In addition, the large amount of LAGB in the bending and straightening samples hinders the dislocation movement during tensile deformation, which has a dislocation strengthening effect. Therefore, the YS and UTS of the bending and straightening plate should be improved when stretched in different directions.

However, during the stretching process of the plate along RD after bending and straightening, the stretching curve has a concave characteristic, and the YS is 141 MPa, which is lower than that of the as-received sample and the sample after annealing. This is because a large number of preset $\left\{10\bar{1}2\right\}$ tensile twins are activated by RD compression of the plate, and the detwinning phenomenon occurs during the reverse stretching of the plate along RD. The process of detwinning is controlled by the migration of twin boundaries [40]. Compared with the process of twin nucleation, the process of migration requires lower energy, that is, CRSS nucleation > CRSS transfer [41]. Furthermore, the detwinning process does not require nucleation, which is attributed to the internal stress generated within the matrix grains during the pre-twinning process [42]. Therefore, the detwinning process can occur at lower stress, resulting in lower yield strength and earlier plastic deformation. As stretching progresses, the grain orientation changes due to detwinning or twinning processes. When the original twins are consumed, the grains change from soft orientation to hard orientation, resulting in a new hardening effect, which is manifested as a rise in the stress–strain curve and an increase in UTS.

It should be noted that the YS stretched along TD is much higher than that stretched along RD. This is because, in the bending and straightening samples, the plate is compressed along RD, causing the c-axis of the grains to be deflected by 86.3° in the RD. At this time, in the {0001} pole figure, the texture intensity in RD is higher than that in TD (Figure 3d), and twinning has less “softening” effect on tensile strength along TD. At the same time, when stretching along the TD direction, it is approximately perpendicular to the de-twining direction, so it is difficult for de-twining to occur. The presence of pre-twins is similar to the effect of grain refinement, which increases the yield strength.

In addition, twinning and dynamic recrystallization refine the grains. Pre-twinning causes a weakening of the basal texture and rotates the matrix grain orientation to a direction that easily activates more slip. It can be observed from the IGMA analysis that the activation of non-basal slip in twinning and matrix grains increases during the bending and straightening process, especially for pyramidal <c+a> dislocations. The formation of dislocation sources activates more homologous dislocations during subsequent deformation and plays a key role in coordinating the strain along the c-axis of the grain. Finally, the fracture elongation in all three directions increases after bending and straightening.

Table 1 also shows the r-values and the Erishen values for various samples. Among them, the Erishen test results of different plates are shown in Figure 12d–f. It is well known that a smaller r value can prevent the thickness thinning during deformation, which is beneficial to improving the stretch formability of magnesium alloys. The as-received sample and the sample after annealing sample have strong basal textures, and the strain in the transverse direction is controlled by the prismatic <a> slip. The strain in the thickness direction can only be coordinated by the pyramidal <c+a> slip system and $\left\{10\bar{1}2\right\}$ tensile twinning [43], while the former cannot be activated at room temperature and the latter is not easily generated when compressed along the c-axis of the grain [44]. Therefore, the as-received plate and the plate after annealing have poor deformability in the thickness direction, resulting in higher r values and lower IE values, which are 2.5 and 2.87 mm, respectively. Table 2 shows the average value and variance in $\bar{r}$ value and IE value for various samples. The variance in $\bar{r}$ value and IE value of each sample is quite small after many tests, and the test results are representative.

After bending and straightening deformation to preset tensile twins, the coordination of twins and various dynamic recrystallization processes leads to the deflection of the basal plane, which effectively weakens the texture of the basal plane. At the same time, they partially coordinate the deformation along the c-axis (thickness direction) of the original grain and further promote the activation of the slip system. As a result, the r-value of the bending and straightening plate is lower, the $\bar{r}$ value is reduced by 77%, and the IE value is increased by 88% to 4.85 mm.

## 4. Conclusions

1. Both twinning and dynamic recrystallization lead to the deflection of the basal plane, which effectively weakens the basal texture. At the same time, various grain orientations also play a positive role in promoting the activation of the non-basal slip system.
2. Part of the continuous dynamic recrystallization (CDRX) and discontinuous dynamic recrystallization (DDRX) inherit the grain orientation of the parent grains, and the other part has random deviations from the parent grains. The vast majority of twin-ning-assisted dynamic recrystallization (TDRX) can effectively inherit the grain orientation of the parent grain and retain the orientation relationship of the twin.
3. When the parent grains are basal orientation, the dynamic recrystallization that deviates from the grain orientation of the parent grains is beneficial to the weakening of the basal texture. When the orientation of the parent grains is non-basal plane orientation, the formation of dynamic recrystallization that inherits its orientation will promote the increase of non-basal oriented grains and play a further role in weakening the basal texture.
4. After bending and straightening, the combination of pre-set tensile twinning and various dynamic recrystallization processes leads to the deflection of the basal plane. Combined with grain refinement strengthening and dislocation strengthening, the magnesium alloy plate after bending and straightening obtains good comprehensive mechanical properties. In particular, the stamping performance is significantly improved.

## Figures and Tables

**Table 1:** Mechanical properties of various samples.

Samples	YS/MPa			UTS/MPa			FE/%			r-Value			$\bar{r}$ -Value	IE/mm
	RD	45°	TD	RD	45°	TD	RD	45°	TD	RD	45°	TD		
AR	187	150	134	270	253	230	8.1	12.9	6.3	2.76	2.50	8.05	3.95	2.58
AS	166	160	169	270	265	270	13.5	19.3	8.7	1.60	2.27	5.36	2.87	2.87
SS	141	132	175	276	250	280	12.8	19.4	13.8	0.97	0.77	1.13	0.91	4.85

**Table 2:** Average value and variance in $\bar{r}$ value and IE value for various samples.

Samples	Average $\bar{r}$ Value	σ^2^ ( $\bar{r}$ Value)	Average IE Value/mm	σ^2^ (IE)
AR	3.90 ± 0.05	1.70 × 10^−3^	2.43 ± 0.4	5.34 × 10^−2^
AS	2.84 ± 0.05	1.67 × 10^−3^	2.85 ± 0.1	2.17 × 10^−3^
SS	0.93 ± 0.02	3.00 × 10^−4^	4.81 ± 0.1	1.20 × 10^−3^

## Data Availability

The data that support the findings of this study are available from the corresponding author upon reasonable request.
